# Setting performance-based financing in the health sector agenda: a case study in Cameroon

**DOI:** 10.1186/s12992-017-0278-9

**Published:** 2017-08-01

**Authors:** Isidore Sieleunou, Anne-Marie Turcotte-Tremblay, Jean-Claude Taptué Fotso, Denise Magne Tamga, Habakkuk Azinyui Yumo, Estelle Kouokam, Valery Ridde

**Affiliations:** 1 University of Montreal, 7101, avenue du Parc, Montréal, Québec H3N 1X9 Canada; 2 Research for Development International, 30883 Yaoundé, Cameroon; 3 World Bank, Office of Yaoundé, Yaoundé, Cameroon; 4 Agence d’Achat de Performance du Littoral, Douala, Cameroon; 5 Université Catholique d’Afrique Centrale, 11628 Nkolbisson, Yaoundé, Cameroon

**Keywords:** Africa, Agenda setting, Cameroon, Kingdon, Multiple streams approach, Performance-based financing, Health policy, Policy analysis, Policy emergence, Case study

## Abstract

**Background:**

More than 30 countries in sub-Saharan Africa have introduced performance-based financing (PBF) in their healthcare systems. Yet, there has been little research on the process by which PBF was put on the national policy agenda in Africa. This study examines the policy process behind the introduction of PBF program in Cameroon.

**Methods:**

The research is an explanatory case study using the Kingdon multiple streams framework. We conducted a document review and 25 interviews with various types of actors involved in the policy process. We conducted thematic analysis using a hybrid deductive-inductive approach for data analysis.

**Results:**

By 2004, several reports and events had provided evidence on the state of the poor health outcomes and health financing in the country, thereby raising awareness of the situation. As a result, decision-makers identified the lack of a suitable health financing policy as an important issue that needed to be addressed. The change in the political discourse toward more accountability made room to test new mechanisms. A group of policy entrepreneurs from the World Bank, through numerous forms of influence (financial, ideational, network and knowledge-based) and building on several ongoing reforms, collaborated with senior government officials to place the PBF program on the agenda. The policy changes occurred as the result of two open policy windows (i.e. national and international), and in both instances, policy entrepreneurs were able to couple the policy streams to effect change.

**Conclusion:**

The policy agenda of PBF in Cameroon underlined the importance of a perceived crisis in the policy reform process and the advantage of building a team to carry forward the policy process. It also highlighted the role of other sources of information alongside scientific evidence (eg.: workshop and study tour), as well as the role of previous policies and experiences, in shaping or influencing respectively the way issues are framed and reformers’ actions and choices.

## Background

The past 15 years have witnessed unprecedented global attention to health challenges in low and middle income countries (LMICs) [1]. In 2015, 17 sustainable development goals (SDGs) were established for 2030. These global goals aim to ensure healthy lives and promote wellbeing for all [2]. Improving health outcomes through efforts to strengthen health systems has become a priority in many LMICs [3]. This improvement often requires substantial changes in the organization and governance of health systems, in the face of limited human and financial resources.

Financing is at the centre of efforts to improve health and health systems. It is only when resources are adequately, efficiently and equitably mobilised, pooled and spent that all people can enjoy sustained progress towards universal health coverage (UHC) [4]. The Third UN Financing for Development Conference in 2015 let to the adoption of the Addis Ababa Action Agenda (AAAA) [5]. The AAAA and the 2030 Agenda for Sustainable Development help facilitate a new global framework for health financing, necessary to support the momentum of UHC.

The question of how to tackle increasing health needs [6, 7] has compelled a vibrant debate on sustainability, mainly centered on the aspirational goal of UHC and the resources needed to finance such an endeavor in LMICs. In response, there have been concerted efforts in many countries to better align their health financing strategies with the ambitious policy aspirations of UHC [8]. Key to this goal, is to improve the efficiency of financial resources.

Contractual arrangements have been suggested as possible tools to this end [9]. Among these, the performance-based financing (PBF) approach has attracted significant attention as a promising intervention for improving health services delivery in LMICs [10–12]. Under PBF, health care facilities and health care workers have more autonomy and receive financial resources upon taking measurable actions or achieving predetermined performance targets. The emergence of performance-based funding programs is part of comprehensive reform goals that can influence all pillars of the health system [13]. Currently, more than 30 countries in sub-Saharan Africa alone, have introduced (or are in the process of introducing) PBF approaches in their health systems [14].

Recent research suggest the positive impacts of PBF on health service utilization [3–6] and on the quality of care [15, 16]. On the other hand, several theoretical and empirical studies have pointed out possible inconsistent and undesirable effects linked to the introduction of PBF in a complex system such as services distortion, gaming, inequity and dilution of intrinsic motivation [17–21]. While many LMIC countries have adopted PBF initiatives, the majority of studies so far focus on presenting the effects and impacts of this approach, and say little about its implementation process. Moreover, there has been little analysis of how such an approach made it onto national policy agendas.

Despite several published studies on how policy issues gain prominence [22–24], or are periodically re-examined and maintained on an agenda over time [25, 26], there remains a dearth of work on these issues in LMICs [27, 28]. Understanding what comes onto the agenda can help frame the problems and thus the solutions [29–31]. Even if agenda-setting is not a part of implementation, how things come on to the agenda influence what policy is formed and implemented [30].

It has been argued that not enough consideration has been given to policy development processes in the health sector of low-income countries [32]. Attention has been paid to the policy contents, ignoring why and how the reforms emerged and were adopted [33]. Analysis of policy reforms in many LMICs led *Grindle and Thomas* to offer a multivariate framework for understanding the policy emergence in developing countries [34]. The framework focuses specifically on the role of policy leaders in shaping policy agendas, balancing policy options and managing political and bureaucratic challenges of policy reform.

Few studies have attempted to apply policy analysis to understanding PBF initiatives in Sub-Saharan Africa [35–37]. To our knowledge, only two studies from Tanzania [37] and Chad [38] provide any comprehensive analysis of the emergence of PBF onto a national agenda. In Tanzania, the authors discussed the policy process behind the introduction of pay-for-performance in maternal and child health, while in Chad, the study attempted to understand why the PBF scheme failed to move from a pilot to the national policy agenda. However, both studies focused particularly on the interests and the roles of actors, and less on political and policy environments in which the reform happened.

Many scholars have described the introduction of major public policy in terms of the influence of economic growth and democratization [39] or the transition from authoritarianism to democracy [40]. However, these factors taken on their own fail to explain why PBF emerged at national policy in many LMIC when considering the context of these countries.

This study contributes to fill this knowledge gap by examining the policy agenda behind the introduction of PBF program in the context of a developing country such as Cameroon.

## Methods

### Context

In late 2004, the Catholic Organization for Relief and Development Aid (CORDAID), a Dutch non-governmental organisation (NGO) started a small pilot PBF project in Catholic health centers in one diocese (Batouri) in the East Region of Cameroon (Phase I). This was extended to two others dioceses (Bertoua and Yokadouma) in late 2008 in the same region of the country (Phase II). The project ended in 2012 and evaluation of the program in one Diocese (Batouri), although it was very limited, suggested some promising results in terms of enhancing accountability and governance at the level of the health facility [41].

The World Bank and the Government of Cameroon decided in 2008 to launch a PBF Project in four out of ten regions to improve the quality and quantity of health care delivery. The project started in February 2011 in the Littoral Region and was extended to three other regions (East, North-West and South-West) in 2012. The PBF project in Cameroon has been implemented in public, private and faith-based organization (FBO) facilities across 26 health districts in the Littoral, North-West, South-West and East regions of Cameroon, covering a total population of approximately 3 million.

Alongside the implementation of this PBF project, the World Bank conducted an impact evaluation in 3 of the 4 regions (East, North-West and South-West) and a qualitative study that focused on understanding the experiences in the piloting of PBF and analyzing the experiential elements of health service delivery (results have not yet been published). However, as in many other settings, there has been little analysis of the process by which PBF was put on the national policy agenda.

### Study design

Our study relied on an explanatory case study design [42]. The case was defined as the PBF program in Cameroon, from late 2004 to March 2015. The levels of analysis were related to the conceptual framework described in the section below.

### Conceptual framework

Sabatier and Jenkins-Smith [43] argued that a common approach to understanding policy processes is to use the ‘heuristic stages’ that aims to simplify how decisions are made and to present the process as a fluid cycle of stages: agenda setting, policy formation, decision-making/policy adoption, implementation, and evaluation [30]. However, it is a “swirly” process where these stages are interdependent and concurrent, not simply composed of distinct steps [44].

Given the paucity of theoretical and conceptual approaches to the analysis of health policy processes in LMIC, *Walt and Gilson* reviewed a set of health policy papers on agenda setting and tested them against a specific priority-setting framework [45].

**Fig. 1:**
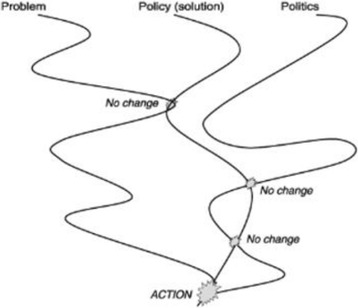
Kingdon’s three stream model of agenda setting. Source: Adapted from *Buse* et al. (30)

Agenda-setting is the first stage in the policy process. The policy agenda consists of moving an idea onto or higher up on that agenda. To guide the policy agenda analysis in Cameroon, we adopted the Kingdon multiple streams framework (Fig. 1) [29]. *Kingdon* proposed to look at this process as a dynamic of three streams: problems, policies, and politics [29].

The problem stream refers to the perceptions of problems as public matters requiring government action and is influenced by previous efforts of government to respond to them [30]. Mechanisms that could make some circumstances problematic include changes in indicators, focusing events, or feedback (e.g., reports or evaluations) [29].

The policy stream consists of the ongoing analyses of problems and their proposed solutions together with debates surrounding these problems and possible responses [30]. The politics stream operates quite separately of the other two streams and is comprised of events such as swings in national politics, changes of government and campaigns by interest groups [30]. In a nutshell, for a policy to become visible on the agenda the following are needed: i) the recognition that a problem should be addressed, ii) a solution that is scalable into a policy, iii) a favorable political environment, and iv) stakeholders to support the policy. According to *Kingdon*, public policies emerge when policy entrepreneurs (i.e. advocates for proposals or for the prominence of an idea) get hold of or create windows of opportunity to couple a problem stream with a politics stream. The convergence of the three streams is initiated by a policy entrepreneur who decides to use these resources to promote convergence at a point where a window of opportunity appears. Entrepreneurs may emerge from any of the streams, depending on the situation and the degree to which a certain stream predominates [46]. Favourable circumstances in all three streams at the same time significantly increase the chance that a policy idea will be placed on the decision agenda.

### Instruments, sample and data collection

This research involved two concurrent qualitative data collection methods.

#### Document review

A document review was important for this study in order to understand the PBF policy in the Cameroonian context. Documents provided background and context, additional questions to be asked, supplementary data, a means of tracking change and development, and triangulation of findings from key informants (KI). Moreover, documents were useful to gather data on events that could no longer be observed or on information that had been forgotten [47].

A total of 35 documents were included in this study. These documents have been instrumental in understanding the policies’ contexts and in supplementing primary data collected from KIs. See Appendix 1 for the list of the documents reviewed.

#### Individual in-depth interviews

The use of interviews provided information that could not be studied in documents. Interviews can illuminate feelings, thoughts, perceptions, and interpretations of the surrounding world as well as the intentions of individual actions [48, 49].

KIs were selected using purposive sampling, with the main criterion being their involvement in the agenda-setting of the PBF program. First, through a brainstorming process, the research team identified all possible institutions and actors who could have had a potential influence on the decision to start a PBF program in Cameroon. We then validated this list with the national coordinator of the PBF project at the Ministry of Public Health (MoPH). From each of the key institutions, the focal persons involved in the policy process were selected. We employed a snowball technique to identify other key respondents until a saturation point. Some of the respondents identified had moved on to other employment or had retired and were categorized under the institutions they were working for at the time of the policy’s emergence. The selected respondents included donor representatives, policy makers, international organization, and researchers.

In total, we conducted 25 in-depth interviews with key stakeholders of the PBF intervention working at various levels: technical and financial partners at international level (*n* = 8), policy makers at the central level, i.e., Ministry of Public Health (*n* = 9), technical and financial partners at national level (*n* = 5) and policy makers at provincial level (*n* = 3). All interviews were recorded.

The objectives of the study were explained to all respondents and written informed consent was obtained before interviews. To ensure confidentiality and anonymity of the data, we replaced participants’ and interviewers’ names with codes.

### Data management and analysis

We conducted thematic analysis [50], guided by our conceptual framework and our knowledge of PBF, to extract the main themes from the documentation and the in-depth interviews. All interviews were transcribed and analyzed using QDA Miner Lite. The coding of data was oriented by organizing the data around conceptual categories. A hybrid deductive-inductive approach allowed us to assign data to predefined themes and to derive new themes from the data.

## Results

The data revealed that the PBF program emerged onto the agenda of policy makers following the coupling of the problems stream (i.e., weak health outcomes, especially for maternal and child health, and inefficiency regarding financial resources) and the politics stream (i.e., national mandates to improve population health), with an important reform of the health sector to achieve the health-related MDGs. This coupling was initiated by actors from the World Bank in cooperation with a network of policy makers (i.e., political entrepreneurs) from the Ministry of Public Health (MoPH). To further our understanding, we describe below the policy agenda into Kingdon’s three streams (problem, politics, and policy) and analyze the role of policy entrepreneurs and windows of opportunities (Table 1).

**Table 1:** Summary of main findings

Problem stream	Politics stream	Policy stream
Key event: National symposium on reproductive health in Yaoundé (1999) Key publication: “*Cameroon country status report: Reversing the Decline in Health Outcomes”* (2003) Recognition of: ➢ poor health outcomes, especially for maternal & child health indicators ➢ inefficiency regarding financial resources, some of which were provided by international donors ➢ highly centralized management of the health system ➢ disjunction in health program implementation between the peripheral & the central level ➢ need for a better health financing policy	The fight against corruption became a high-priority mandate: ➢ Sparrowhawk operation ➢ National Anti-Corruption Commission ➢ prosecution of some officials PBF was framed as a way to fight against corruption Endorsement of PBF approach by newly appointed Minister of Public Health	Loans and grants to implement PBF from the World Bank & the Health Results Innovation Multi Donor Trust Fund program Context of institutional reforms with the adoption of the sector wide approach (SWAP) ➢ an attempt to improve the coordination of the development assistance for health ➢ seen as a tool for improving aid effectiveness by ensuring policies, budgets and institutional arrangements, and to improve the sectoral performance. Existence of a contracting process within the health sector that started in the early 2000s.
Policy entrepreneurs		
Work Bank representatives acted as policy entrepreneurs by: ➢ initiating a policy dialogue with decision makers from the Ministry of Public Health, through meetings and presentations of evidence from Rwanda ➢ initiating & supporting the participation of a delegation of officials from the Ministry of Public Health in a PBF study tour in Rwanda A network of PBF entrepreneurs influenced the position of international bilateral agencies (E.g.: GIZ) regarding PBF Entrepreneurs had good communication, lobbying and networking skills as well as important political connections.		
Windows of opportunity		
Windows of opportunity were created which contributed to pushing PBF higher onto the agenda: ➢ a series of meetings & international workshops on PBF ➢the deadline of the MDGs and the government’s priority to achieve these MDGs ➢ other countries in Sub-Saharan Africa were adopting PBF ➢ a reform of public finance marked a shift from an input-based budget to a results-based budget in many sectors such as health and education the Ministry of Public Health made PBF a high priority within the Implementation of the Health Sector Support Investment Project (HSSIP), under the SWAP		

### Problem stream

The main challenges faced by the Cameroon health system can be summarized in five points: (i) the high level of out-of-pocket payments for patients, (ii) the low quality of care, (iii) the difficult regulation of a growing private for-profit sector, (iv) the lack of qualified human resources, and (v) the lack of accountability [51, 52].

Despite huge investments in health by many funders, health indicators in Cameroon were stagnating, especially for maternal and child health. There was therefore a need for an innovative approach that could improve the health of the population as highlighted by the following quotes.

*“I think that the main idea for the World Bank was that for so many years, so many donors had put so much money into the health system and, despite all that, the changes are not that big, eg the maternal and child mortality rate got even worse here in Cameroon. So, it was obvious that Cameroon had to change direction in order to meet its global engagements” (*Policy maker, provincial level MoPH).

The problems regarding poor maternal and child health outcomes had been recognized by the state even earlier than 2000. Approximately ten years ago, Cameroon was confronted with high rates of maternal and child mortality. The rates increased from 454 deaths per 100,000 live births in 1996 to 669 deaths per 100,000 live births in 2004 [53]. In 2005, the former Minister of Health, Mr. Urbain Olanguena Awono, acknowledged that the maternal mortality rate was unacceptable and that it was necessary for the government of Cameroon to react by drawing up an action plan based on the principles of equity, social justice and national solidarity [54].

We traced an important event around the end of the 1990s that contributed to understanding the issue of poor maternal health outcomes as an important problem. During the National Symposium on the Reproductive health held in Yaoundé from the 14th to the 17th December 1999, many national experts and policy makers expressed their concerns about the worsening of maternal and child outcomes. This led the country to define eight priorities sectors taking into account national specificities [55].

The publication of a report [56] by the World Bank in June 2003 drew further consideration to this issue. It advocated that the time had come to pay greater attention to poor maternal and child health status. In fact, the publication of the report entitled “*Cameroon country status report: Reversing the Decline in Health Outcomes*”, was a key element in the broader recognition of the poor health status as a real problem that required attention. The importance of this report was highlighted during our interviews and respondents indicated that this important document had a large influence on understanding the health sectors’ problems in Cameroon.

*“There was a report on the health system – a Country status report - that the Bank released; the Bank produces a sectoral report every five years for each sector. It’s more or less a descriptive analysis of the sector, what are the weaknesses, strengths, challenges, etc. and there was one in 2003-2004, that was not very good. But this report guided the CMU (Country Management Unit) to provide an IDA (International Development Association) grant to support the health sector”* (Official, International Organization).

The above-mentioned report revealed a difficult paradox: the disconnect between socioeconomic indicators and health indicators. The results of this evaluation showed that health indicators were not proportionate with the level of wealth of the country. Above all, it was striking to notice that some countries (e.g. Lesotho) with socioeconomic levels well below that of Cameroon had much better health outcomes. There were allocative efficiency issues, in that the majority of public funds for health care were allocated to the central and provincial levels of the health care system, and peripheral levels with the greatest need were receiving the least funds, as some KIs put it:

*“The infant mortality rate was very high compared to some countries in the same or even lower level of wealth as Cameroon. And we also knew that many women and children were dying due to the lack of adequate care” (*Policy maker, central level MoPH).

The analysis also revealed two other political/strategic disconnects: the highly centralized management of the health system and a real disjunction in health program implementation between the peripheral and the central level. It was striking to see that at the central level, high-level discussions were taking place, but that nothing concrete was translating to the operational level. In this regard, one respondent confided:

*“The one thing that always struck me was the level of sophistication and the quality of the policy debates and policy discussions. And then, when you look at the implementation on the ground -and I visited many hospitals and health centers and so forth-, there was a disconnect again”* (Official, International Organization).

The publication of the World Bank report provided evidence on the state of health outcomes and health financing, and raised awareness of the situation. Through the aforesaid report, this international organization developed measures that marked the severity of the health system problem in the country, and made political leaders aware of these measures so they could not plausibly deny that a problem existed. As a result, the decision-making level came to the conclusion that the lack of a suitable health financing policy to produce good outcomes was an important issue (in the problem stream) that needed to be addressed by the government.

### Politics stream

Within the orientation stream of the Cameroon PBF program, a series of background mandates and reforms allowed the introduction and adoption of the PBF program.

Firstly, the fight against corruption came in as a high-priority mandate for the government. Indeed, in the late 90s, the ranking of Transparency International reported twice that Cameroon had the greatest perceived corruption index [57]. An informant highlighted the corruption issue in by the following quote:

*“My recollection is that there were frustrations that were very typical of Cameroon eh, corruption, mismanagement, etc” (*Official, International Organization).

*“So much money has been pumped into the system […]. I can tell you that the per capita health financing in Cameroon, I think it was around or more than $USA 60! But it still gave nothing! The indicators ... if you see Cameroon’s MDG eh! Maternal mortality keeps increasing. Governance was not at the top” (*Policy maker, provincial level MoPH).

In 2004, the government launched the Sparrowhawk operation (i.e: Name given to the vast judicial operation for the fight against corruption in Cameroon) to put the management of public funds in order. In October, 2004, the President stressed in an election campaign speech that the fight against corruption would be a priority for his government if he was re-elected. After his re-election, actions were taken to fulfil this promise.

In 2005, the Cameroonian President declared: *“... I have given instructions to the Government to put the battle up a notch ... We cannot fight against poverty by letting people divert public funds”* [58].

Then in 2006, following Cameroon’s classification as a heavily indebted poor country initiative (HIPC), his determination to improve the governance of the country became more apparent by intensifying and densifying the tone of his speech: *“those who have enriched themselves at the expense of the public fortune must disgorge ... white collar offenders had better watch out”* [58].

In this context, any initiative or program that supported the fight against corruption tended to be well-received. For example, there was the creation of the National Anti-Corruption Commission in 2006 (NACC). The PBF initiative also gained prominence within this context, as one policy maker put it:

*“It should be said that our presentation aimed to frame PBF to show that it could be used to fight against corruption and for the efficient financial management of the health system’s resources....” (*Policy maker, central level MoPH).

The political changes, including the cabinet reshuffle in December 2007, which led to a new Minister of Public Health in an environment where the government was committed to the fight against corruption and for which several senior officials including several ministers (for example the former ministers of public health and finance) and general directors suspected of embezzlement and illegal enrichment were prosecuted, gave an additional impetus to the reflections. In the following months, officials with great political and technical reputations, as well as considerable experience, were appointed to the administrative inspection services of the Ministry of Public Health, with a mandate to pursue reforms that were underway in the health system. Later, one of these officials was designated to coordinate the PBF project management unit at the time of its implementation. The newly appointed Minister of Public Health’s endorsement of the PBF program was then fundamental to push the policy onto the agenda, as one informant expressed:

*“ the Minister of Public Health was very inspired and motivated, you know, to do something in this area. I think we have to give them credit”* (Official, International Organization).

All political discourse was converging towards greater accountability to the population, and also to a more efficient health system. The changes in the political stream supported the emergence of a policy initiative that favoured the introduction of new political ideas on PBF.

### Policy stream

The original Cameroon Health Sector Support Investment Project funded by the World Bank was a five-year US$25 million project (US$20 million District Service Delivery + US$5 Institutional Strengthening). It received the World Bank Board approval in May, 2008, and became effective in March 2009. The project underwent a restructuring in June, 2011, and again in March 2014, with a revised end date of January, 2016 [59].

Moreover, the 25 million dollars for PBF initiatives from the World Bank served as a catalyst for the political impetus in the agenda-setting of the project in Cameroon. Interviews with some respondents who played key roles in setting the PBF in the agenda give us more insight:

*“It has to be said first of all that this project has been the signing of a credit agreement No. 4478CM of... 25 million dollars, that’s $ 25 million to support this form of innovative financing strategy” (*Policy maker, central level MoPH).

This loan came within the context of the concessional rate loans programs of the International Development Association (IDA) at first, then later as a grant from the Health Results Innovation Multi Donor Trust Fund program (HRITF), a multi-donor fund created by the World Bank in 2007, with funding from Norway and the UK, to support the development of health-related PBF, as one official noted it:

“*Cameroon was a little advanced. Cameroon had a credit in place before the Trust Fund was set up. That is to say that for many countries, the Trust Fund served as lever. In Cameroon, it is the credit that served as lever*” (Official, International Organization).

The idea was also fertilized by the context of institutional reforms within the health system, with the adoption of the sector wide approach (SWAP), an attempt to improve the coordination of the development assistance for health, where we also noted the involvement of high-level players. The SWAP was seen as a tool for improving aid effectiveness by ensuring policies, budgets and institutional arrangements likely to lead to improvements in sectoral performance. There was the implementation of a health sector support investment project (HSSIP), under the SWAP. This opened a window of opportunity and prompted the Ministry of Public Health to make the PBF project a high priority within the HSSIP framework.

Moreover, a contracting approach between the public and the private sector had already been proposed as a solution in order to improve the performance of health systems. In this approach, the MoPH was engaged in a contractual relationship with the confessional health system [60].

The process of contracting (also used in the PBF approach) within the health sector actually started in the early 2000s where conditions were progressively put in place for its development: a collaboration framework (2001), a health sector strategy (2001–2010), the appointment of a sub-director in charge of national partnership (2002), and the gradual convergence of several partners around the Division of Cooperation (DCOOP) of the MoPH on the need to develop a global partnership approach [60]. The process was accelerated by the inception of the debt relief contract project, which provided a mandate to support the private not-for-profit sector through contracting. The work of drafting a partnership strategy was begun in 2003 and ended in 2006 [60].

### Policy entrepreneurs

Data suggests that it is the World Bank that carried the idea of ​​developing PBF. The PBF program in Cameroon included several prominent policy entrepreneurs who played important roles in agenda-setting. These included officials from the World Bank and the Ministry of Health.

Some senior officials from the World Bank were working in both Rwanda and in Cameroon. They saw at that time, through the early and encouraging results from Rwanda [11], an opportunity to introduce the same strategy in Cameroon. Therefore, these players from the World Bank initiated a policy dialogue with decision makers from the Ministry of Public Health, through meetings and presentations of evidence from Rwanda, to consider how Cameroon could adopt such an approach. The interview with one respondent who played key role in this regard give us more insight:

*“Once I did a presentation you know at the Ministry of Public Health in Yaoundé about the Rwanda experience, and there were various other things that the World Bank was recognizing is part of this global movement of value for money. So, when we put the health project together, we thought that this, you know, testing out this approach will be really a good thing in the Cameroonian context”* (Official, International Organization).

Although there were recognised problems, as mentioned above, as well as policy mandates / directives, the PBF program was not on the agenda of policy makers until the end of 2007, when the World Bank initiated and supported the participation of a delegation of officials from Cameroon’s Ministry of Public Health in a PBF study tour in Rwanda. Thanks to promising results that were showcased during the study tour, these officials, who can be seen as political entrepreneurs, came back very motivated and acted as catalysts for change. They succeeded in coupling the two streams of problems and policies. The importance of this study tour is pointed out by the following extract:

*“Already when we came back I think in 2007 from Rwanda, we made a presentation to all the officials in the Ministry. What they said was that we were enthusiastic and that it was not sure that what Rwanda was doing would succeed in Cameroon”* (Policy maker, central level MoPH).

The network of experts from the World Bank and officials from the MoPH played a crucial role in persuading, through lobbying, the government to consider the PBF as a program of high priority and importance. These policy entrepreneurs used encouraging results from Rwanda’s PBF program to claim that Cameroon should change its current ineffective health financing mechanism and embrace a performance-based approach. They tried to convince high-level authorities at both the MoPH and the Ministry of Finance. These entrepreneurs, who were determined to place PBF on the agenda of policy makers, had good communication, lobbying and networking skills and also had important political connections. They used their skills to persuade all influential officials to join the development of the PBF program.

For instance, the network pushed the endorsement of the PBF program by the newly appointed Minister of Public Health and this was fundamental to bring the policy into the agenda setting. The network also worked to influence the position of some international bilateral agencies (E.g.: GIZ), who were at the time against the introduction of the PBF approach [61]. In addition, they organized workshops and meetings to generate widespread attention to the issue, and presented leaders with policy alternatives, so that policymakers came to believe problems could be surmounted and that they knew what they were expected to do.

The presence of political entrepreneurs was an important factor in opening windows of opportunity to political innovation by linking the three streams of problems, politics and policy.

#### Windows of opportunity

A series of meetings and international workshops contributed to push PBF higher onto the agenda.

First of all, most of the key informants evoked the close deadline of the MDGs and the government’s priority to achieve these MDGs.

*“I had an opportunity to go to Sierra Leone where I represented Cameroon in a meeting on the MDGs uh ... so we were about twenty-five, fifteen countries and realized that those who had started with the PBF had quickly improved their MDGs” (*Policy maker, central level MoPH).

We should note that apart from Rwanda, many other countries in Sub-Saharan Africa like Burundi, Democratic Republic of the Congo, Tanzania and Zambia were implementing or adopting PBF approach, and were seen as flagship countries or innovators in such reforms. This increasing momentum for PBF adoption fostered space for mutual forms of knowledge exchange and learning activities. In other words, transnational advocates positioned the PBF policy as a South-South learning process open to all countries willing to embark on it.

In the national context, the adoption of the PBF program coincided with the reform of public finance laws. Largely driven by the government, the reform was a public symbol of the presidents’ commitment to transparency. This was marked by a shift from an input-based budget to a results-based budget in many sectors such as health and education. Some players recognized this as an element that weighed considerably for the emergence of the PBF approach in Cameroon.

*“Public finances were governed by the law of 1962 that evolved, and then in 2007, there is the 2007/2008 law of December 26* ^*th*^ *, 2007 that reforms public finances in Cameroon, changing the budget from a means-based to a results-based budget”* (Policy maker, central level MoPH).

The results above suggest that the PBF program benefited from a dynamic series of national and international meetings, study tours in other countries, and an ongoing health system as well as public finance reforms to become embedded on the agenda of policy makers.

## Discussion

The study aimed to investigate how PBF was placed on the policy agenda in Cameroon. Unlike user fee exemption policies that have been driven by national policies makers [62], results showed that the PBF agenda in Cameroon was driven by a global player (i.e The World Bank), raising questions regarding the principle of sovereignty and right of national actors to make their own policy choices [63]. Global health players, including funding agencies, are key components of the policy making process in many LMICs [64], and the quality of their policy advocacy influences the degree to which the issue receives policy attention. Our findings show that this global player generated the interest of national health officials and shaped the degree to which performance-based financing emerged on the national policy agenda through numerous forms of influence, each identified in previous research on agenda setting [65–70].

The first form of influence was financial. The results showed that readily available loan of 25 million dollars from the World Bank served as a monetary window of opportunity and a catalyst for the political impetus in the agenda setting of the PBF project in Cameroon. International relations scholars have identified resource provision, like the financial and technical assistance from international institutions, as a mechanism to generate the interest of national health officials to embrace a particular cause [65, 66].

The second form of influence refers to ideation. Ideas are internal and external frames, or ways in which actors portray and position issues to resonate with audiences [67]. Scholars have shown the major role of ideas in shaping policy change [68, 71–73]. As an alternative view, *Schmidt* points out that ideas can also open a window of opportunity for policy change as the discourse is able to shift actors’ preferences on a policy problem [74]. Ideas can become powerful ideological instruments that actors can use to challenge existing institutional arrangements [75]. Through framing processes, they can help convince policy makers and interest groups that reform is essential [74]. Relating *Schmidt’s* view to our case study, international PBF advocates from the World Bank employed numerous discourses to persuade national policy makers of the importance of this reform. The Bretton Woods institutions defined the development challenges in Africa as a crisis of governments’ inability to manage national affairs, or of governance [76], and argued for a new development paradigm based on good governance [77]. This increasing good governance discourse of the health system opened the door for some changes in Cameroon, making it possible to interpret PBF as a tool that could enhance accountability and efficiency of the health system.

Finally, the officials from the World Bank relied on network- and knowledge-based forms of influence [69]. Their network used several levers to influence policy decision-making, especially “leverage politics,” which is the ability to solicit more powerful stakeholders to influence a situation [70].

Even if the 2004 pilot PBF program of CORDAID did not influence the agenda setting of the PBF program of the Cameroon government [78], it contributed to developing a pool of national PBF experts who collaborated with SINAHEALTH, one of the global PBF pioneers and champions, to develop the PBF reference manual [79] and the Cameroon PBF international training course [78]. This expansion of PBF knowledge in Cameroon was also useful to push the policy in the agenda. In addition, the World Bank actors built their network with these national experts and presented Cameroon at the international level as a leading PBF country. These windows of opportunities (i.e., international and national), appeared over many years during the process, suggesting that the policy agenda might be reached through an incremental process [80, 81].

Our findings showed how the three streams of problem, politics and policy coupled at the agenda-setting stage and provide four lessons about policy emergence.

First, it emphasizes the importance of a perceived crisis in policy reform and supports findings from prior public policy research [32, 34]. In such a situation, there is high demand for reform and fast action; the stakes are perceived significant; transformation is viewed as innovative and high-level policy-makers are involved in the policy reform [34]. In Cameroon, the high level of corruption [57] was perceived as a crisis and the fight against it came in as a high-priority mandate for the policy leaders. In addition, there was high government interest in the outcomes of MDGs 4 and 5, since maternal mortality was showing rising trends, contrasting with the significant reduction (49% reduction) that Sub-Saharan Africa was experiencing at the same time [82, 83]. This marked lack of progress in health outcomes relating to MDGs 4 and 5 in Cameroon put great pressure on the Government to find a way to improve things in order to reach international targets in child and maternal health. We argue that the possibility of failing to meet these targets could also be interpreted as a crisis situation. This echoes past research in Ghana, where the agenda-setting of the NHIS reforms were such that there was a strong perception of a crisis and a need for change among political, technical and bureaucratic decision makers and civil society [32]. In a nutshell, these crisis environments in Cameroon served as a source of power for policy actors to influence the PBF as a policy agenda item. We therefore agree with *Erasmus and Gilson* [84] that power is at the heart of health policy processes, as this case study shows how policy entrepreneurs used contextual features as power leverage to validate their actions and choices.

The second lesson emphasised by our study is the importance of building a team to carry forward the policy process. Multiple streams approach positions policy entrepreneurs and their strategies at the heart of policy change [85, 86]. In Cameroon, having a new pool of technocrats with good communication, lobbying and networking skills as well as important political connections in the MoPH, as the lead in policy development served as an important factor in opening windows of opportunity towards the introduction of the PBF program onto the national agenda. Strategies and actions of particular policy entrepreneurs, principally leaders who can change the distribution of power to facilitate reform, are at the heart of successful health reform [87]. In other words, health reform leadership requires political ability and judgment, in addition to political motivation and commitment.

The third lesson of this case study is that historical policies and experiences can shape the actions and choices of reformers. Path dependency, simply put that the past has a powerful effect on the present, is a strong theme in health policy [88, 89]. For example, *Pisani* et al. (2017) demonstrate that Indonesia’s comprehensive health insurance scheme reflects an incremental evolution from prior health care policies as well as the imprint of deeper historical origins [90]. The team of policy entrepreneurs who led the policy change in Cameroon were all informed by the past experience of the unsuccessful policy initiative of the original sector wide approach. Actions to apply performance-based funding approaches were driven by the perceived failure of traditional input-oriented funding to achieve much progress towards international targets such as the Millennium Development Goals in LMICs [91]. If a country already has policies reflecting pro-market reforms such as public-private partnership initiatives, as was the case in Cameroon with the contracting approach between the public and the private sector, the new policy might emerge more easily [91]. There were some recognized problems such as centralized resources and a lack of a strategic purchasing approach to health, for which PBF appeared as a promising solution/policy. The stream of politics was very dominant at this stage as the solution/policy was consistent with the national mandates/directives of good governance.

A final policy lesson from this study is the lack of scientific evidence in the policy emergence. Despite the fact that scientific evidence was pivotal in bringing the problem stream into the attention of policy makers, it played a limited role amidst the other main sources of information, that include key events, study tour and workshops, in shaping or influencing the way the issue was framed [29, 45].

Largescale happenings such as conferences and workshops that attract the attention of key stakeholders also have agenda-setting power [92]. This is consistent with the finding that policy and decision-making processes are not normally underpinned by the scientific evidence [93, 94].

It is important to note some limitations of this analysis. Though we draw some lessons from the Cameroon PBF program, we recognize the limitations of a single case study such as researcher subjectivity and construct validity [95], and highlight the necessity for comparative case analysis to improve the generalizability of these lessons beyond the Cameroon case. Moreover, some actors were unavailable for interviews; the missing data is of greater concern for the policy analysis, where missing information could result in a lack of understanding of the process, content and actors’ role in the agenda setting. However, we sought to mitigate this potential bias by combining multiple sources of data.

For our case study, the findings demonstrate the utility of the Multiple Streams Model as (i) a lens and guide for data collection, and (ii) a means to organize and analyse the data. However, we also concur with *Shroff et al*. that the Kingdon’s framework does not permit the analysis of the boundaries and the relative importance of each of the three streams [87]. For example, the engagement to meet MDGs played a role in both the politics and problem streams. In addition, we have seen that the fight against corruption in the political arena had a major impact on how the policy stream developed. In so doing, the findings of this study indicate that further research should look at how the streams interact and influence each other. Finally, when applying the Kingdon multiple streams framework, it was not clear how to make the distinction between the governmental agenda and the decision agenda [38], and to understand the thinking behind the policy process [96]. Future research could also look at this.

## Conclusion

Transnational actors are key components of the policy making process in many LMICs, and the quality of their policy advocacy influences the degree to which the issue receives policy attention. They generated the interest of national health officials and shaped the degree to which the policy innovation emerged on the national policy agenda through numerous forms of influence (financial, ideational, network and knowledge based).

The Cameroon case underlines the importance of a perceived crisis in a policy reform and the advantage of building a team to carry forward the policy process. It also highlighted the role of other sources of information alongside scientific evidence, as well as the role of previous policies and experiences, in shaping or influencing respectively the way an issue is framed and actions and choices of reformers.

## Appendix 1

### List of the documents reviewed

1. AEDES. Performance Based-Financing implementation procedures manual. AEDES: Brussels; 2012.
2. Brenzel L and Schneidman M. Results-based financing at the world bank - Cameroon country snapshot 54082. WB: Washington DC; 2009.
3. CORDAID. Internal evaluation of the performance-based financing project in the East region of Cameroon. CORDAID: The Hague; 2014.
4. CORDAID. Next step for the PBF in the East region of Cameroon January-March 2015. Proposal to the Ministry of Public Health Cameroon. CORDAID: Bertoua; 2013.
5. Health Sector Support Investment Project (HSSIP). Impact evaluation of two years of PBF on quality and use of health services in the Littoral region – Cameroon. HSSIP: Yaounde; 2013.
6. Health Sector Support Investment Project (HSSIP). Note of the HSSIP-World Bank joint mission, August 23 to September 14, 2012. HSSIP: Yaounde; 2012.
7. Health Sector Support Investment Project (HSSIP). Report of the quarterly Performance Purchasing Agencies meeting from December 17 to 19, 2012, in Douala. HSSIP: Yaounde; 2012.
8. Health Sector Support Investment Project (HSSIP). Report of the third quarterly Performance Purchasing Agencies meeting from July 17 to 18, 2013, in Bertoua. HSSIP: Yaounde; 2013.
9. Health Sector Support Investment Project (HSSIP). Report of the Workshop on Strengthening the Capacity of Fiduciaries of the Regions and Production of the Second Quarter 2013 Financial Monitoring Report, Ebolowa (NKOLANDOM) from August 2 to 7, 2013. HSSIP: Yaounde; 2013.
10. Integrated Safeguards Data Sheet Additional Financing. Report No.: ISDSA7312. WB: Washington DC; April 2014
11. Keugoung B, Tsafack JP, Ymele Fouelifack F, Sieleunou I, Ayissi Noubosse I. Performance Based-Financing pilot project in the diocese of Batouri in Cameroon: lessons for the extension of the model. CoP PBF: Antwerp; 2011.
12. Kimanuka C and Taptue JC. Report of the baseline Household Survey and the baseline Quality Survey for the Performance Based-Financing Program in 4 Health Districts: Cité des palmiers, Edea, Loum and Yabassi, 2011-2014, In comparison with the control Districts : Nylon, Mbanga, Melong, Logbaba, Nkongsamba, Manjo. Littoral Regional Fund for Health: Douala; January – February 2011.
13. Le Mentec R, Mettling C. Regional Funds for Health Promotion : Operation - Strengths - Challenges. GIZ: Yaounde; 2014.
14. Littoral Regional Funds for Health Promotion (RFHP). Administrative, Financial and Accounting Procedures Manual for the Implementation of the Performance Based Financing Project in the Littoral Region. RFHP: Douala; November, 2011.
15. Messen B., Antony M. Report of the research support mission, strategical and technical monitoring. AEDES: Brussels; 2012
16. Ministry of Public Health (MoPH). Contrat of services No 0114/CS/MINSANTE/PAISS/12-2011 between the Ministry of Public Health and AEDES. MoHP: Yaounde; 2012.
17. Ministry of Public Health (MoPH). Contribution of the MoPH to the development of Cameroon - Programmatic report 2009-2013. MoPH: Yaounde; 2009.
18. Ministry of Public Health (MoPH). Decision No : 0118/D/MINSANTE/CAB of March 13, 2011, on consultation and kit treatment. MoPH: Yaounde; 2012.
19. Ministry of Public Health (MoPH). Decision No 1483/D/MINSANTE/CAB/PAISS of December 24, 2014, transferring the management of the North-West Performance Purchasing Agency to the North-West Regional Funds for Health Promotion. HSSIP: MoPH; 2014.
20. Ministry of Public Health (MoPH). Decision No: 0032/MINSANTE/CAB/ of January 24, 2011 on health care exemption policy. MoPH: Yaounde; 2011.
21. Ministry of Public Health (MoPH). Roadmap for the reduction of maternal and neonatal mortality in Cameroon 2006 – 2015. MoPH: Yaounde; 2006.
22. National Institute of Statistics. Demographic Health and Multiple Indicators Survey. INS: Yaounde; 2011.
23. Soeters R. Support Mission for the Health Sector Support Investment Project (HSSIP), Performance Based Financing program in Cameroon, from August 25 to September 14, 2012. HSSIP: Yaounde; 2012.
24. Soeters R.et Enandjoun B. Report of the World Bank Mission on Performance Based Financing pilot project from October 19 to 31, 2009. The World Bank: Yaounde; 2009.
25. Sorgho G. Cameroon RBF Operation: Technical Design Matters! World Bank: Kigali, Rwanda; Jun 27, 2010.
26. Sorgho G. Report of the mid-term evalaution of the Health Sector Support Investment Project (HSSIP) from May 6 to 17, 2013. HSSIP: Yaounde; 2013
27. The World Bank. Cameroon Country Status Report Reversing the Decline in Health Outcomes. WB: Washington DC; June 2013.
28. The World Bank. Cameroon Economic Update Towards Greater Equity A Special Focus on Health. WB: Washington DC; July 2013.
29. The World Bank. Cameroon economic update. Towards better service delivery an economic update on Cameroon. WB: Washington DC; July 2011. Issue No.2.
30. The World Bank. Implementation Status & Results. Cameroon Health Sector Support Investment (SWAP) (P104525). WB: Yaounde; December 2013.
31. The World Bank. Implementation Status & Results. Cameroon Health Sector Support Investment (SWAP) (P104525). WB: Yaounde; June 2013.
32. The World Bank. Implementation Status & Results. Cameroon Health Sector Support Investment (SWAP) (P104525). WB: Yaounde; May 2014.
33. The World Bank. Building Evidence on Results-Based-Financing (RBF) for Health: Third Annual Impact Evaluation Workshop. World Bank: Bangkok, Thailand; Oct 17, 2011.
34. The World Bank. Toward greater equity. Cah Économique Cameroun. World Bank: Washington DC; 2013.
35. UNFPA. Why invest in reproductive health in Cameroon. UNFPA: Yaounde; 2012.
