# Erratum to: ‘Setting performance-based financing in the health sector agenda: a case study in Cameroon’

**DOI:** 10.1186/s12992-017-0288-7

**Published:** 2017-08-21

**Authors:** Isidore Sieleunou, Anne-Marie Turcotte-Tremblay, Jean-Claude Taptué Fotso, Denise Magne Tamga, Habakkuk Azinyui Yumo, Estelle Kouokam, Valery Ridde

**Affiliations:** 1 University of Montreal, 7101, avenue du Parc, Montréal, Québec, H3N 1X9 Canada; 2 Research for Development International, 30883 Yaoundé, Cameroon; 3 World Bank, Office of Yaoundé, Yaoundé, Cameroon; 4 Agence d’Achat de Performance du Littoral, Douala, Cameroon; 5 Université Catholique d’Afrique Centrale, 11628 Nkolbisson, Yaoundé, Cameroon

## Erratum

After publication of the article [1], it has been brought to our attention that the analytical categories used in the first part of the discussion section, developed by Lara Gautier [2], was not cited. Furthermore, work of Barnes et al. [3] is now cited in the paragraph related to the PBF policy.

The authors would like to update the following paragraphs to refer to her work [2].

1. We should note that apart from Rwanda, many other countries in Sub-Saharan Africa like Burundi, Democratic Republic of the Congo, Tanzania and Zambia were implementing or adopting PBF approach, and were seen as “flagship countries” [2] or innovators in such reforms.
2. In other words, transnational advocates positioned the PBF policy as a “South-South learning” [3] process open to all countries willing to embark on it.
3. Our findings show that this global player generated the interest of national health officials and shaped the degree to which performance-based financing emerged on the national policy agenda through numerous forms of influence, each identified in previous research on agenda setting [4–9] **and have been conceptualized by Gautier** [2].
4. The first form of influence was financial [2].
5. The second form of influence refers to ideation [2].
6. Finally, the officials from the World Bank relied on “network-*”* [2] and “knowledge-based*”* [2] forms of influence [9].

## References

[1] Sieleunou I, Turcotte-Tremblay A-M, Fotso J-CT, Tamga DM, Yumo HA, Kouokam E, et al. Setting performance-based financing in the health sector agenda: a case study in Cameroon. Glob Health. 2017;13:52. https://doi.org/10.1186/s12992-017-0278-9. 10.1186/s12992-017-0278-9 PMC5540528 28764720

[2] Gautier L. From ideas to policymaking: exploring the diffusion of performance-based-financing at the global, continental, and national levels: case study in Mali. Research Protocol. University of Montreal; 2016.

[3] Barnes A, Brown GW, Harman S. Introduction: Global Politics of Health Reform in Africa. In: Global Politics of Health Reform in Africa: Performance, Participation, and Policy. London: Palgrave Macmillan; 2015. p. 1–18. doi:10.1057/9781137500151_1.

[4] Shiffman J. Generating political priority for maternal mortality reduction in 5 developing countries. Am J Public Health. 2007;97(5):796–803. doi:10.2105/AJPH.2006.095455. 10.2105/AJPH.2006.095455 PMC1854881 17395848

[5] Diaz Pedregal V, Destremau B, Criel B. Health policy in Cambodia: to what extent is an aid-dependent country able to determine its own policy? J Soc Policy. 2015;44(01):171–87. doi:10.1017/S0047279414000543.

[6] Lapping K, Frongillo EA, Studdert LJ, Menon P, Coates J, Webb P. Prospective analysis of the development of the national nutrition agenda in Vietnam from 2006 to 2008. Health Policy Plan. 2012;27(1):32–41. doi:10.1093/heapol/czr013. 10.1093/heapol/czr013 21330309

[7] Anderson E. Experts, ideas, and policy change: the Russell Sage Foundation and small loan reform, 1909–1941. Theory Soc. 2008;37(3):271–310.

[8] Parkhurst JO, Vulimiri M. Cervical cancer and the global health agenda: insights from multiple policy-analysis frameworks. Glob Public Health. 2013;8(10):1093–108. doi:10.1080/17441692.2013.850524. 10.1080/17441692.2013.850524 PMC3877944 24236409

[9] Robert E, Ridde V. Global health actors no longer in favor of user fees: a documentary study. Glob Health. 2013;9:29. doi:10.1186/1744-8603-9-29. 10.1186/1744-8603-9-29 PMC3750575 23889807

